# Effect of Growth Temperature on the Structural and Electrical Properties of ZrO_2_ Films Fabricated by Atomic Layer Deposition Using a CpZr[N(CH_3_)_2_]_3_/C_7_H_8_ Cocktail Precursor

**DOI:** 10.3390/ma11030386

**Published:** 2018-03-05

**Authors:** Jong-Ki An, Nak-Kwan Chung, Jin-Tae Kim, Sung-Ho Hahm, Geunsu Lee, Sung Bo Lee, Taehoon Lee, In-Sung Park, Ju-Young Yun

**Affiliations:** 1 School of Electronics Engineering, Kyungpook National University, Daegu 41566, Korea; js-0102@kriss.re.kr (J.-K.A.); shhahm@knu.ac.kr (S.-H.H.); 2 Materials and Energy Measurement Center, Korea Research Institute of Standards and Science (KRISS), Daejeon 34113, Korea; cnk@kriss.re.kr (N.-K.C.); kimjt@kriss.re.kr (J.-T.K.); 3 Department of Nanomaterials Science and Engineering, University of Science and Technology, Daejeon 34113, Korea; 4 Eugene Technology Materials, Gyeonggi-do 16675, Korea; gslee@egtmaterials.com; 5 Department of Materials Science and Engineering and Research Institute of Advanced Materials, Seoul National University, Seoul 08826, Korea; bolee@snu.ac.kr; 6 Division of Materials Science and Engineering, Hanyang University, Seoul 04763, Korea; dlxogns1218@hanyang.ac.kr; 7 Institute of Nano Science and Technology, Hanyang University, Seoul 04763, Korea

**Keywords:** cocktail precursor, atomic layer deposition, ZrO_2_, CpZr[N(CH_3_)_2_]_3_/C_7_H_8_, capacitor

## Abstract

The effect of growth temperature on the atomic layer deposition of zirconium oxide (ZrO_2_) dielectric thin films that were fabricated using a CpZr[N(CH_3_)_2_]_3_/C_7_H_8_ cocktail precursor with ozone was investigated. The chemical, structural, and electrical properties of ZrO_2_ films grown at temperatures from 250 to 350 °C were characterized. Stoichiometric ZrO_2_ films formed at 250–350 °C with an atomic ratio of O to Zr of 1.8–1.9 and a low content of carbon impurities. The film formed at 300 °C was predominantly the tetragonal crystalline phase, whereas that formed at 350 °C was a mixture of tetragonal and monoclinic phases. Electrical properties, such as capacitance, leakage current, and voltage linearity of TiN/ZrO_2_/TiN capacitors fabricated using the thin ZrO_2_ films grown at different temperatures were compared capacitor applications. The ZrO_2_ film grown at 300 °C exhibited low impurity content, predominantly tetragonal crystalline structure, a high dielectric permittivity of 38.3, a low leakage current of below 10^−7^ A/cm^2^ at 2 V, and low-voltage linearity.

## 1. Introduction

Zirconium oxide (ZrO_2_) thin films have been extensively studied as dielectrics and insulators because they exhibit a high dielectric constant (k), acceptably low leakage current, and good thermal stability [1]. These favorable properties have led to commercial use of ZrO_2_ thin films as dielectrics and insulators in a variety of microelectronics applications, such as dynamic random access memory [2], radio-frequency and analog/mixed-signal (RF/AMS) integrated circuits [3,4], and non-volatile resistive random access memory [5].

The crystalline structure of ZrO_2_ films strongly influences their dielectric and electrical properties. The reported static k values of ZrO_2_ are 19.7, 46.6, and 36.8 for monoclinic, tetragonal, and cubic phases, respectively [6], and the respective bandgap energies (Eg) are in the ranges of 5.16–7.09, 5.78–6.62, and 6.1–7.08 eV [7,8,9]. When compared with the monoclinic phase, the tetragonal and/or cubic phases have higher k and similar Eg, which result in the benefits of a ZrO_2_ dielectric film that is thinner with higher capacitance.

Because the crystalline phase of ZrO_2_ depends on the growth process parameters, several methods, such as using an oxidant and doping with another element, have been used to obtain tetragonal ZrO_2_ films [10,11,12]. The addition of an oxidant like H_2_O or O_3_ during atomic layer deposition (ALD) determines the crystallographic structure of the resulting ZrO_2_ film [10]. ZrO_2_ films grown at 300 °C with H_2_O are a mixture of monoclinic, tetragonal, and cubic phases. In contrast, films grown with O_3_ contain little of the monoclinic phase. Even when ZrO_2_ films are subjected to rapid thermal annealing at 800 °C, they mostly retain their crystalline phases, with just a marginal increase of the content of the monoclinic phase. While the ZrO_2_ films that were grown with H_2_O keeps the k value of 24 even after RTA, he k value of the film grown with O_3_ is enhanced from 25 to 30 after RTA. Impurity doping of ZrO_2_ films is another method used to stabilize the tetragonal phase, which is thermodynamically unstable at low temperature. A tetragonal/cubic ZrO_2_ metastable phase has been obtained by doping ZrO_2_ with Si or Ge impurities with small ionic radii [11]. In addition, Fe-doped ZrO_2_ thin films fabricated at 350 °C contained the tetragonal/cubic ZrO_2_ phase [12].

ALD has been widely used to fabricate ZrO_2_ films for use as capacitor dielectrics [3,13]. ALD has been used to fabricate highly conformal coatings of various metal oxide films, such as SiO_2_, Y_2_O_3_, and HfO_2_, with thicknesses of the order of several to a hundred nanometers on complex three-dimensional capacitor structures with a high aspect ratio [14,15,16]. In the ALD process of a binary metal oxide, the film grows through the surface reaction of a metal precursor and oxidant that are alternately supplied to and purged from the underlying substrate. Each pulse step of the precursor and oxidant should be well isolated to prevent any gas-phase reactions between them.

Suitable choice of a metal precursor is crucial for a successful ALD process [17]. The metal precursor used for film growth requires sufficiently high vapor pressure, purity, and thermal and chemical stability, along with low viscosity and cost. A CpZr(NMe_2_)_3_/C_7_H_8_ cocktail precursor with low viscosity and high vapor pressure that meets these requirements has been reported [3]. Using this precursor, a high growth rate of 0.8–0.9 Å/cycle has been obtained over a wide temperature range of 250–400 °C. The ALD growth temperature strongly affects the physical, chemical, and electrical properties of the resulting film. In particular, the growth temperature needs to be regulated to ensure that the maximum k value is obtained for films intended for capacitor applications. Controlling the growth temperature is a very simple method in order to obtain the high-k tetragonal phase of ZrO_2_ without any additional doping or annealing processes.

In this study, the influence of the ALD growth temperature on the chemical, structural, and electrical properties of ZrO_2_ films is systemically investigated. ZrO_2_ films are grown by ALD at 250 to 350 °C. The chemical and structural properties of the films are investigated by X-ray photoelectron spectroscopy (XPS), X-ray diffraction (XRD), and transmission electron microscopy (TEM). The electrical properties of the ZrO_2_ films are obtained from capacitance–voltage (C–V) and current–voltage (J–V) measurements using metal–insulator–metal (MIM) capacitors. The suitability of the filmsfor RF/AMS applications is evaluated. The ability of the growth temperature to regulate the crystalline phase of the films to enhance k without any tradeoff of the leakage current and capacitance linearity is discussed.

## 2. Materials and Methods

The ZrO_2_ dielectric films were prepared by ALD using CpZr(NMe_2_)_3_/C_7_H_8_ as the metal precursor and O_3_ as an oxidant [3]. The CpZr(NMe_2_)_3_/C_7_H_8_ cocktail precursor was vaporized at 65 °C, and then introduced into the reaction chamber using Ar carrier gas through a gas line maintained at 100 °C. O_3_ was produced from high-quality O_2_ (>99.999%) by an O_3_ generator. The ZrO_2_ layers were prepared at different growth temperatures in the range of 250–350 °C. The ALD cycle consisted of pulsing with CpZr(NMe_2_)_3_/C_7_H_8_, purging with Ar gas, pulsing with O_3_, and purging with Ar gas.

The ZrO_2_ dielectric films were grown on Si and TiN substrates. The thicknesses of the ZrO_2_ films on Si substrates were measured with a spectroscopic ellipsometer (Horiba, UVISEL) and those on TiN were confirmed by TEM (FEI, Tecnai G2 F30). The chemical compositions and impurities in the bulk ZrO_2_ films were checked by XPS (Thermo Fisher Scientific, Theta Probe AR-XPS) after removing the surface layer contaminated with carbon and –OH or water using 10-kV Ar-ion etching for 10 min. The crystalline structure of the films was investigated using grazing-incidence XRD (Rigaku, SmartLab). Diffraction patterns were also obtained by fast Fourier transform (FFT) of the cross-section of high-resolution TEM images of the ZrO_2_ films.

To measure the electrical properties of the ZrO_2_ films, MIM capacitors were fabricated. As a bottom electrode, TiN was deposited on the Si substrate by DC sputtering. The TiN top electrode was structured by conventional optical lithography and lift-off to give square pads of 100 × 100 μm. C–V and I–V measurements were performed to evaluate the dielectric and electrical properties of the MIM capacitors using a LCR meter (HP 4284) and a semiconductor parameter analyzer (HP, 4155). The capacitance was measured by sweeping the top electrode voltage from +2 to −2 V, and then vice versa with a small AC signal at 1 kHz. 

## 3. Results and Discussion

**Figure 1:**
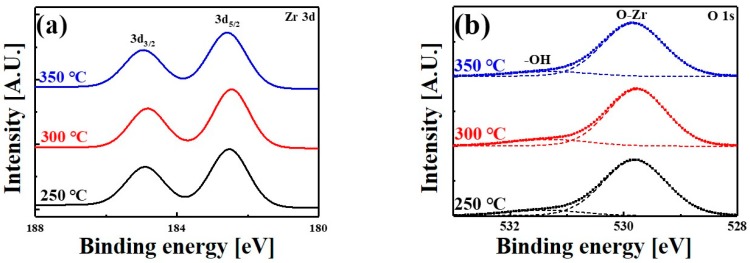
(**a**) Zr 3d and (**b**) O 1s spectra of ZrO_2_ films grown at different temperatures.

Figure 1 shows the Zr 3d and O 1s spectra of ZrO_2_ films grown at 250, 300, and 350 °C. The extracted positions of the doublets and spin-orbit splitting (SOS) of the Zr peaks are summarized in Table 1. The Zr 3d spectrum of the ZrO_2_ film prepared at 300 °C featured two main spin-orbit doublets at 182.46 eV (3d_5/2_) and 184.83 eV (3d_3/2_) (Figure 1a), and are in good agreement with the XPS spectrum of ZrO_2_ [18,19]. The 3d_5/2_ and 3d_3/2_ peak positions of the three films were very close, and the SOS was very similar in the range of 2.37–2.38 eV. These results mean that the chemical state of the ZrO_2_ films remained very similar, regardless of the growth temperature. The O 1s spectra showed two peaks around 530 and 531 eV, which were assigned to zirconium oxide and hydroxyl groups, respectively (Figure 1b). The intensity of the peak around 531 eV decreased with increasing growth temperature. 

**Figure 2:**
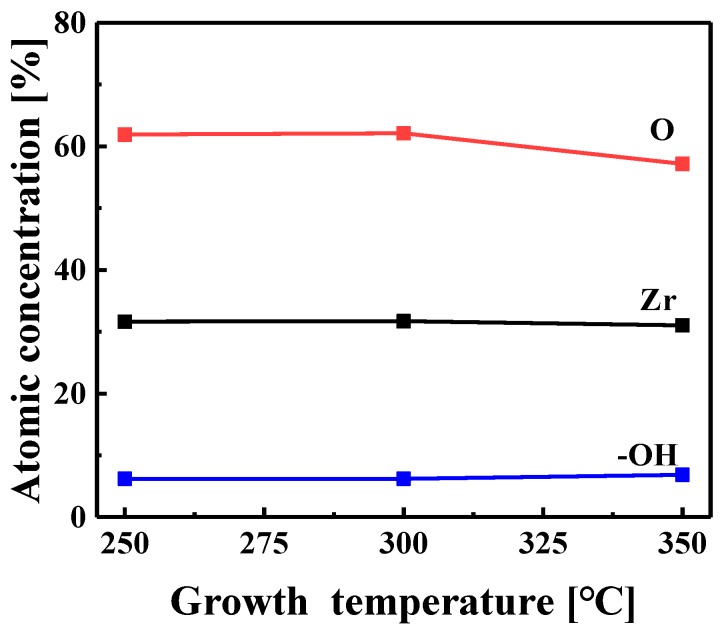
Compositions of ZrO_2_ films grown at different temperatures.

**Figure 3:**
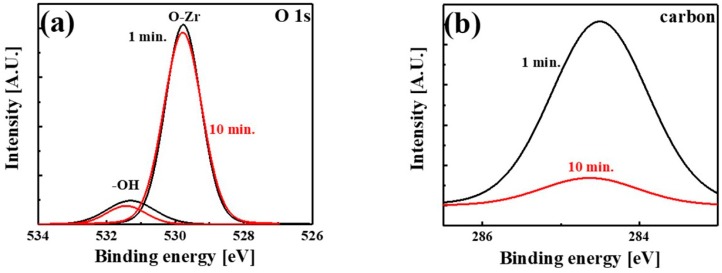
(**a**) O 1s and (**b**) carbon spectra of the ZrO_2_ film grown at 300 °C. The measurements were performed after the film was etched for 1 and 10 min to analyze the composition of the film at the surface and in the bulk, respectively.

Figure 2 shows the composition of the ZrO_2_ films deposited at various temperatures. The ratio of O to Zr was 1.96, 1.96, and 1.84 for the films grown at 250, 300, and 350 °C, respectively. The films grown at 250 and 300 °C were nearly stoichiometric, whereas that grown at 350 °C was oxygen-deficient. There were some –OH and carbon impurities in the ZrO_2_ films. The films contained approximately 6% –OH, as shown in Figure 2. To compare the –OH and the carbon impurity contents of the surface and bulk of a ZrO_2_ film, XPS analyses conducted at different depth profiles of the ZrO_2_ film grown at 300 °C are shown in Figure 3. The ZrO_2_ films were etched for 1 or 10 min and then XPS measurements were performed to obtain surface and bulk data, respectively. The peak ratio of –OH to O–Zr was very similar regardless of the etching time (Figure 3a), which indicated the intrinsic presence of –OH during the growth process or the extrinsic penetration of water into the bulk of the film. In contrast, the intensity of the carbon impurity peak decreased markedly as the etching time lengthened, suggesting the surface contamination of the film by carbon during air exposure. The carbon content of the ZrO_2_ film etched for 10 min was as low as the XPS detection limit, whereas that of the film etched for 1 min was approximately 2%. 

**Figure 4:**
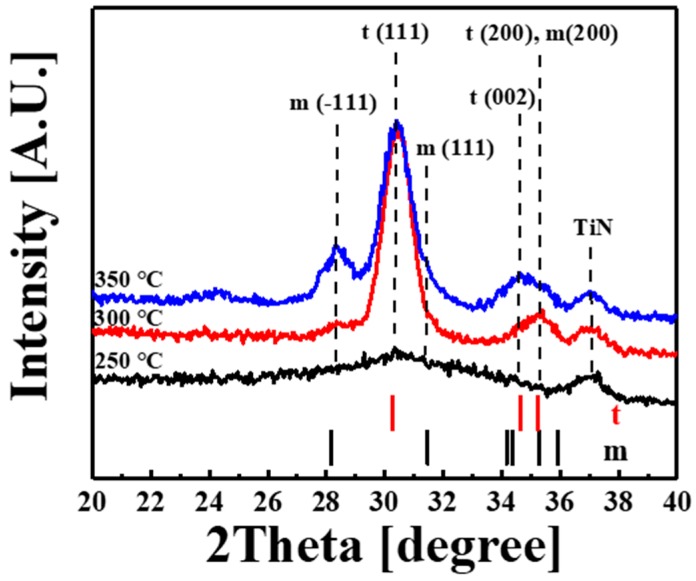
XRD patterns of crystalline ZrO_2_ films grown on TiN substrates at different temperatures. The peaks assigned to tetragonal (t) and monoclinic (m) phases are indicated.

Figure 4 shows the XRD patterns of the ZrO_2_ films grown at 250–350 °C on TiN substrates. For the film that is grown at 250 °C, a small broad peak at 2θ = 30.4° was observed, which was attributed to the tetragonal (111) reflection (JCPDS 14-0534). The ZrO_2_ films grown at both 300 and 350 °C were polycrystalline with their XRD patterns containing peaks at 28.3°, 30.4°, 31.3°, 34.7°, and 35.3°. Regardless of the growth temperature, the dominant peak appeared at 30.4° and was consistent with the tetragonal phase. The other small peaks observed at 28.3° and 35.3° were, respectively, indexed as monoclinic (−111) (JCPDS 36-420) and a mixture of the tetragonal (200) and monoclinic (200) reflections. From the asymmetric peak around 35.3°, a very small peak at 34.7° consistent with tetragonal (002) was extracted. As the growth temperature was increased to 350 °C, the peaks at both 28.3° and 34.7° became much more prominent and another peak appeared at 31.3° on the edge of the dominant peak at 30.4°, which was assigned to monoclinic (111) and indicated the presence of the monoclinic phase. The XRD results are summarized in Table 2. From these results, one can conclude that the tetragonal phase began to appear at 250 °C and was the dominant phase formed at 300 °C.

On the other hand, the crystallite size of the ZrO_2_ films was determined from the peak appeared at 30.4° using by Scherrer formula, $D=k_{1}\lambda ⁄Bcos\theta$, where *k_1_* is the shape factor, which has a numerical value of 0.9; *λ* is the wavelength of the XRD measurement used; *B* is the full width at half maximum of the measured peak in radians; and, *θ* is the Bragg angle [20]. The ZrO_2_ films grown at 300 and 350 °C showed *D* values of 7.4 and 7.3 nm, respectively, which are similar to each other.

**Figure 5:**
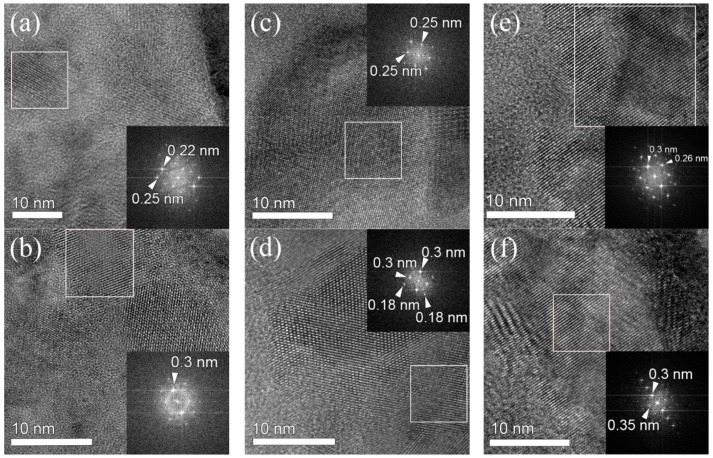
TEM images of ZrO_2_ films grown at (**a**,**b**) 250 °C, (**c**,**d**) 300 °C, and (**e**,**f**) 350 °C. Inset are corresponding Fourier transforms obtained from the areas indicated by squares.

Figure 5 shows TEM images of the ZrO_2_ films and their Fourier transforms extracted from regions inside the squares in the TEM images. The TEM images showed that all of the films were polycrystalline. It is found that the crystallites with several nm in diameter are randomly distributed in the films and are surrounded by amorphous phases, regardless of the film growth temperature. FFT images were obtained from two regions for each film. Both the tetragonal and/or monoclinic phases with spacing d values of 0.30 and 0.25–0.26 nm, respectively, as consistent with those calculated from the XRD peaks at 35.3° and 30.4°, were observed for all the films. The *d* value of 0.18 nm obtained for the film grown at 300 °C could also be assigned to the tetragonal or monoclinic phase. For the films grown at 250 and 350 °C, the calculated *d* values of 0.22 and 0.35 nm were attributed to the monoclinic phase of the ZrO_2_ films. These results strongly supported those of XRD; that is, the tetragonal phase of ZrO_2_ appeared at 250 °C and became dominant at 300 °C, whereas the monoclinic phase coexisted with the tetragonal phase in the film grown at 350 °C.

The phase of ZrO_2_ films strongly depends on the metal precursor, oxidant, film thickness, and growth temperature. The crystalline phases of ZrO_2_ films grown with the CpZr(NMe_2_)_3_ precursor were investigated. The thick ZrO_2_ films grown with CpZr(NMe_2_)_3_ and O_3_ at 250 and 300 °C exhibited only the cubic phase, whereas that grown at 350 °C showed a mixture of the cubic and monoclinic phases [21]. The thick ZrO_2_ films that were grown with CpZr(NMe_2_)_3_ and H_2_O at temperatures from 200 to 400 °C contained mixtures of the tetragonal, cubic, and monoclinic phases [22]. The maximum intensity of the dominant peak of the tetragonal phase at 30.5° was obtained for the film grown at 250 °C and the small intensity of the peak at 28.2° from the monoclinic phase remained constant for films grown at 200 to 300 °C. These findings are consistent with our results; in particular, the dominant peak was observed at approximately 30.4°, regardless of whether it was assigned to tetragonal or cubic ZrO_2_ [3]. 

**Figure 6:**
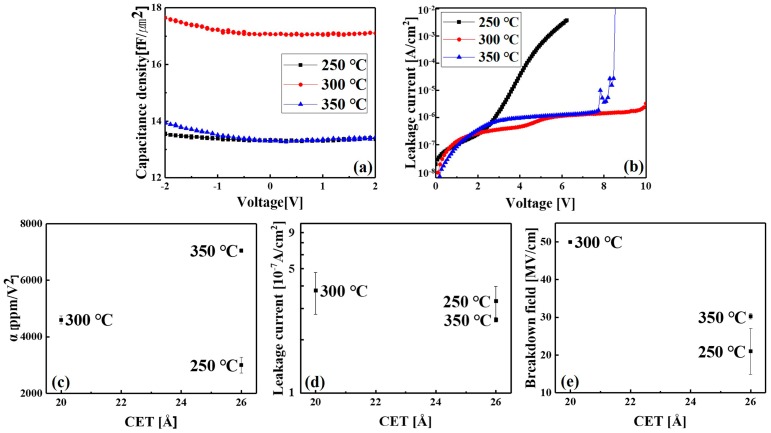
(**a**) Normalized capacitance and (**b**) J–V curves of TiN/ZrO_2_/TiN metal-insulator-metal (MIM) capacitors containing ZrO_2_ films deposited at different growth temperatures. (**c**) Log–log relationship between the quadratic voltage coefficient (α) and capacitance density in the MIM capacitors. Comparison of (**d**) leakage current density and (**e**) breakdown field as a function of capacitance equivalent thickness. For the J–V measurements, the applied voltage was up to 10 V; breakdown occurred over 10 V in the case of 300 °C.

The MIM capacitor used as a passive element in RF/AMS integrated circuits requires a high capacitance density, low leakage current density, and low voltage linearity [20]. The C–V and J–V curves measured for the MIM capacitors with ZrO_2_ insulating dielectrics grown at 250, 300, and 350 °C are shown in Figure 6a,b, respectively. As shown in Figure 6a, the C–V curves depended on both the growth temperature of ZrO_2_ and voltage. The J–V curves were similar below 3 V, but then displayed different shapes over 3 V and different breakdown voltages. For the J-V curve of the MIM capacitor containing the film that was grown at 300 °C, voltage breakdown occurred over 10 V. The voltage linearity, leakage current, and breakdown voltage of the capacitors evaluated from the C–V and J–V curves are redrawn, according to the capacitance equivalent thickness (CET) in Figure 6c–e, respectively.

To evaluate CET, *C*_0_ was capacitance obtained at 0 V from the C–V curves in Figure 6a and then CET was determined using the equation $\mathrm{CET}\;=\;A\;\times \;\varepsilon_{SiO_{2}}/C_{0}$, where A is the effective area of the capacitor and $\varepsilon_{SiO_{2}}$ is the k value of SiO_2_. CET was 26, 20, and 26 Å for the ZrO_2_ dielectric films grown at 250, 300, and 350 °C, respectively. The physical thickness of ZrO_2_ ($t_{ZrO_{2}})$ between TiN electrodes evaluated from the TEM images was 15.2, 20.0, and 18.4 nm for the films grown at 250, 300, and 350 °C, respectively. The dielectric constant of ZrO_2_ ($\varepsilon_{ZrO_{2}}$) for the films grown at 250, 300, and 350 °C was 22.8, 38.3, and 27.6, respectively, as determined from the relationship $\varepsilon_{ZrO_{2}}=\;t_{ZrO_{2}}/CET\;\cdot \;\varepsilon_{SiO_{2}}$ using the above thickness values. The highest $\varepsilon_{ZrO_{2}}$ value of the ZrO_2_ film grown at 300 °C is attributed to its high content of the tetragonal phase. The films that were prepared at 250 and 350 °C had lower $\varepsilon_{ZrO_{2}}$ values because they contained lower contents of the tetragonal phase.

As shown in Figure 6a, the voltage-dependent C–V curves could be described by the second-order equation *C*(*V*) = *C*_0_(*αV*^2^ + *βV* + 1), where *α* and *β* are quadratic and linear voltage coefficients, respectively. Figure 6c shows the relationship between *α* and CET for the ZrO_2_ films grown at different temperatures. Supposing that *α* exhibits an inverse linear relationship with physical thickness for the same film [3,23], the result for the ZrO_2_ film grown at 300 °C is beneficial to obtain a lower quadratic coefficient with a thinner film. The leakage currents obtained at 2 V are presented with respect to CET in Figure 6d. The lower the CET of the dielectric film, the higher the leakage of the capacitor. However, for device applications, a lower leakage current with a thinner dielectric is required. The ZrO_2_ film grown at 300 °C showed the advantageous property of a higher capacitance and similar or lower leakage current (below 10^−7^ A/cm^2^ at 2 V) when compared with those of the other ZrO_2_ films. Finally, a high breakdown voltage is required for a dielectric to remain stable under electrical stress. The breakdown field of the devices plotted against CET is shown in Figure 6e. The dielectric ZrO_2_ film grown at 300 °C exhibited a higher breakdown field than those of the other films, despite having a lower CET.

## 4. Conclusions

We investigated the structural and electrical characteristics of ZrO_2_ thin films grown by ALD using the CpZr[N(CH_3_)_2_]_3_/C_7_H_8_ cocktail precursor and O_3_ at various temperatures. The ZrO_2_ films that were grown at 250–300 °C were stoichiometric with negligible carbon contamination. The crystalline phases of the films varied with growth temperature. Predominantly tetragonal phase and a mixture of tetragonal and monoclinic phases were obtained at 300 and 350 °C, respectively, which matched well with the measured dielectric and electrical characteristics. The capacitance density, leakage current, and breakdown voltage of MIM capacitors containing the ZrO_2_ thin films were determined. A low leakage current and high dielectric constant were realized for the ZrO_2_ film grown at 300 °C because of its high content of the tetragonal phase. The leakage current density of the TiN/ZrO_2_/TiN MIM capacitors was approximately 10^−7^ A/cm^2^ at 2 V. The capacitance density was related to the growth temperature. In particular, the MIM capacitor containing a ZrO_2_ film that was grown at 300 °C displayed a high dielectric permittivity of 38.3 because of its high content of the tetragonal phase. 

## Figures and Tables

**Table 1:** Zirconium (Zr) three-dimensional (3d) peak positions and spin-orbit splitting (SOS) of zirconium oxide (ZrO_2_) films grown at different temperatures.

Temperature (°C)	3d_5/2_ (eV)	3d_3/2_ (eV)	SOS (eV)
250	182.51	184.89	2.38
300	182.46	184.83	2.37
350	182.57	184.94	2.37

**Table 2:** Intensities of XRD peaks corresponding to monoclinic (m) and tetragonal (t) phases observed for ZrO_2_ films fabricated at different growth temperatures.

Temperature (°C)	28.3° (m)	30.4° (t)	31.3° (m)	34.7° (t)	35.3° (t, m)
25	-	low	-	-	-
30	low	high	-	low	medium
35	medium	high	Low	medium	low
